# Knowledge of Physicians about the Interrelationship between Diabetes Mellitus and Periodontitis in the United Arab Emirates

**DOI:** 10.1055/s-0042-1746413

**Published:** 2022-07-11

**Authors:** Khawla Al Matrooshi, Sireen Al Raeesi, Abdel R. Tawfik, Amar H. Khamis, Crawford Bain, Momen Atieh, Maanas Shah

**Affiliations:** 1 Department of Periodontology, Ministry of Health and Prevention, United Arab Emirates; 2 Department of Oral Surgery, Mohammed Bin Rashid University of Medicine and Health Sciences, Hamdan Bin Mohammed College of Dental Medicine Dubai Healthcare City, Dubai, United Arab Emirates; 3 Department of Biostatistics, Mohammed Bin Rashid University of Medicine and Health Sciences, Hamdan Bin Mohammed College of Dental Medicine Dubai Healthcare City, Dubai, United Arab Emirates; 4 Department of Periodontology, Mohammed Bin Rashid University of Medicine and Health Sciences, Hamdan Bin Mohammed College of Dental Medicine Dubai Healthcare City, Dubai, United Arab Emirates; 5 Department of Oral Diagnostics and Surgical Sciences, Mohammed Bin Rashid University of Medicine and Health Sciences, Hamdan Bin Mohammed College of Dental Medicine Dubai Healthcare City, Dubai, United Arab Emirates; 6 Sir John Walsh Research Institute, Faculty of Dentistry, University of Otago, Dunedin, New Zealand; 7 Department of Periodontology, Mohammed Bin Rashid University of Medicine and Health Sciences, Hamdan Bin Mohammed College of Dental Medicine Dubai Healthcare City, Dubai, United Arab Emirates

**Keywords:** diabetes, interrelationship, knowledge, periodontitis, United Arab Emirates

## Abstract

**Objective** Diabetes mellitus is a major cause of morbidity in the United Arab Emirates (UAE), highlighting a significant social and economic burden impacting the development of the country. Studies have shown a bidirectional relationship between diabetes and periodontal disease. The awareness of this relationship is imperative not only for dentists but also for the physicians who contribute toward enhancing a diabetic patient's health and lifestyle. There is a general need to highlight the importance of maintaining periodontal health and its positive effect on controlling diabetic health status. The purpose of this study is to investigate the knowledge of the physicians regarding diabetes and periodontal health.

**Materials and Methods** A cross-sectional study involving a questionnaire was distributed to the physicians who were attending the Arab Health Conference in Dubai in 2019.

**Statistical Analyses** A cross-tabulation analysis compared attitude, knowledge, and awareness across sector, gender, and country of graduation. A logistic regression model was used to explain the “knowledge” regarding possible confounding factors.

**Results** A total of 344 physicians with an average age of 38.11 (9.31) years, comprising of 186 (53.8%) males, participated in the survey. Of those participants, 285 (82.8%) were working in the government sector health care centers versus the private health care organizations. Also, 108 (31.4%) of the participants graduated within the universities based in the UAE, while the remaining 263 (68.6%) participants graduated from medical schools from other countries. At least 265 (77%) of the participants had positive outlook toward referring patients with diabetes to their dental colleagues, while 283 (82.3%) of the physicians acknowledge that diabetes affects periodontal health. While a majority of participants 261 (76%) treat diabetic patients in their clinical practice, only 50 (19%) of the participants admit to referring these patients for a dental consultation. In addition, the survey also revealed that 225 (65.5%) physicians comprehend the bidirectional relationship of periodontal disease and diabetes.

**Conclusion** An appropriate dental referral protocol is recommended for all diabetic patients who visit physicians. This survey demonstrated that although the physicians present with good knowledge, they rarely refer diabetic patients to receive proper periodontal care.

## Introduction

Diabetes mellitus (DM) is a global threat that affects all countries, regardless of its socioeconomic status. Over the past few years, the United Nations (UN) and World Health Organization (WHO) have encouraged countries to take cognizance of increased morbidity and mortality rates due to diabetes. Despite this, very few countries have an established national diabetes plan. The International Diabetes Federation (IDF) presented epidemiological data compiled from 138 countries in its latest edition. 1 A prevalence of 9.3% of the world population (∼463 million adults from ages 20–79 years) suffer from diabetes and its related complications. This total number is forecasted to increase to 700 million (10.9%) by 2045. In addition, the mortality rate resulting from diabetes and its complications has been estimated to be 4.2 million in 2019. 1 More specifically, comparative prevalence of diabetes in a population adjusted for 20 to 79 years of age is the highest in the Middle East/North Africa (MENA) region. (12.2% in 2019 and estimated 13.9% in 2045). More locally, there are various studies evaluating the prevalence of diabetes and prediabetes in the United Arab Emirates (UAE). 2 3 4 The Dubai Health Authority (DHA) in collaboration with the Dubai Statistics Center, conducted the Dubai Household Health Survey (DHHS) in 2014 and additional supplementary data on undiagnosed diabetes and prediabetes were published in 2017. 5 A remarkably high prevalence of diabetes (overall: 15.2%) was noted among the UAE nationals (19%) and expatriate population (14.7%). Furthermore, a large section of diabetic and prediabetic population was left undiagnosed (10.8% of the participants involved in the survey). The study also highlighted modifying contributing factors that may have impacted the prevalence of diabetes in the Emirati population. 5 In addition, the socioeconomic impact of diabetes-related health care expenditure cannot be underestimated. The financial burden associated with diabetes within the MENA region totaled U.S. dollar (USD) 24.9 billion which is expected to be increased by 55% to USD 38.6 billion in 2045. 6

The bidirectional association between periodontal disease and DM is well documented. 7 The presence and accumulation of periodontal pathogens produce a variety of virulence factors that play a significant role in initiation and progression of biofilm-induced periodontal disease. 8 Furthermore, the immune host response also plays a fundamental role in enhancing periodontal tissue destruction. This chronic inflammatory process triggered by the bacterial insult and its reactive host response further worsens in presence of comorbidities like diabetes. 9 An increased level of periodontal destruction characterized by elevated levels of proinflammatory cytokines (interleukin [IL]-1-β, tumor necrosis factor-α, IL-6, etc.) has been observed in poorly controlled diabetes. 10 There is a strong consensus that suggests the bidirectional relationship between these diseases. However, the precise mechanism connecting diabetes and periodontal disease has not been fully elucidated yet. Studies have suggested the presence of a distinct subgingival microbial profile and variation in the immune response for diabetic patients. 11 12 The role of receptor for advanced glycation end products (RAGE) in inducing a hyperinflammatory response to periodontal infection is highlighted in various studies. 13 14 15 The hyperglycemic state observed during diabetes induced expression and activation of RAGE. Furthermore, increased inflammation and oxidative stress can contribute to advanced glycation products (AGEs) formation. Initial expression of advanced glycation products (AGEs) and markers of oxidative stress were demonstrated in gingival tissues of diabetic patients with periodontitis. 14 Subsequently, increased serum levels of AGEs in patients with increased periodontal destruction in type-2 diabetic adults has also been observed. 16 Severe periodontal disease has, reciprocally, shown to unfavorably affect blood glucose levels in patients with or without confirmed diabetes. Stage-III and -IV periodontitis patients have been associated with poor glycemic control and an increased risk of development of diabetes. 10 A dose-dependent relationship between the conditions has also been established and stability of periodontal health is important to minimize the ill effects of periodontitis on diabetes and its complications. 17

This two-way relationship mandates frequent communication between medical physicians and dental professionals to achieve optimum well-being for a diabetes patient. The International Diabetes Federation (IDF) also recommends implementing good oral health as a part of diabetes management by medical care professionals. 6 Since a diabetes patient, typically, is examined and treated at a medical center, it is important to gauge the knowledge and attitude among physicians to direct their diabetic patients for their periodontal health evaluation and possible management. There is scarce evidence within the region that has evaluated the awareness of the physicians in this matter.

The main aims of this study were to investigate the knowledge of medical physicians, working in public and private sector within the UAE, regarding the interrelationship between periodontal diseases and DM, along with their attitude toward involving dental professionals in overall management of their diabetic patients.

## Materials and Methods

### Study Design and Criteria for Selection

This study utilized a cross-sectional methodology, based on a structured self-designed questionnaire that was assembled by investigators for addressing the main aim of the study. The survey was pretested by conducting a pilot study and the feedback of the participants was deliberated by the authors. Prior to the initiation of the study, ethical approval was obtained from the Institutional Review Board Committee, and the study was conducted in full conformance with ethical principles highlighted by the “Declaration of Helsinki”. 18 A hard-copy printed questionnaire with a cover letter was prepared and distributed among medical physicians attending the Arab Health Conference in Dubai in 2019. The physicians, willing to participate voluntarily, were provided with a verbal informed consent at the beginning of the survey highlighting the aims and objectives of the questionnaire. There was no financial incentive offered. The survey was anonymous and could be collected at the end of the day from the participants. Any incomplete responses or unfinished data from the survey data were excluded from the study for further analysis. There was no follow-up with nonrespondents due to the anonymity of the survey.

### Study Survey

The survey formulated in English language, consisted of 20 questions; six items covered the demographical characteristics of the participants, seven questions investigated the physician's knowledge regarding the susceptibility of diabetic patients to periodontal disease, three questions evaluated their attitude toward need for appropriate dental care procedures for diabetic patients, and five questions were related to awareness of interrelationship between periodontal disease and DM. The questionnaires were answered anonymously in an unlimited time.

### Statistical Analysis

Descriptive statistics was used to give a clear demonstration of the sample. A cross-tabulation analysis was used to compare attitude, knowledge, and awareness across sector, gender, and country of graduation. Comparison of categories of knowledge by age, years of graduation, score of attitudes, and score of awareness were also achieved statistically. A logistic regression model was used to explain the “knowledge” regarding possible factors (i.e., age, gender, sector, attitude, and awareness of the participants).

## Results

Of the total 550 surveys distributed, 344 physicians, 185 (53.8%) males with an average age of 38.11 ± 9.31 years, were completed and returned the questionnaire ( Table 1 ). Surveys supplying with incomplete data were excluded from the analysis (37.5% excluded from the total distributed surveys). Also, 82.8% of the participants, completing the survey, were associated with medical practice in government-based health care centers. One hundred eight participants (31.4%) had graduated from within the UAE, while the remaining 263 participants (68.6%) graduated from medical schools based in other countries. The number of years lapsed after graduation and years of experience for these participants were 13.47 ± 8.88 and 12.80 ± 8.89 years, respectively ( Table 1 ).

**Table 1:** Demographic characteristics of the participants

Item	No. (%)
Surveys distributed	550 (100)
Surveys collected at the end of day	420 (76.3)
Surveys with complete data	344 (62.5 of total distributed)
Gender	
Male	185 (53.8)
Female	159 (46.2)
Sector	
Public	285 (82.8)
Private	59 (17.2)
Country of graduation	
Others	263 (68.6)
UAE	108 (31.4)
Age (y) Mean (SD)	38.11 (9.31)
Years since graduation Mean (SD)	13.47 (8.88)
Years of experience Mean (SD)	12.80 (8.89)

Abbreviations: SD, standard deviation; UAE, the United Arab Emirates.

### Exposure to Diabetes Patients and Referrals

The descriptive statistics results demonstrated that 255 (74%) of the participants claimed to see more than 50 diabetes patients per year, while 48 (14%) of participants examined between 25 and 50 diabetes patients per year, and 41 (12%) of the participants encountered up to 25 diabetes patients per year. The study also revealed that a majority of the participants (76%) never referred their diabetic patients to a dentist, and only 19% of them referred less than 20% of their diabetes patients to a dentist. A minimal 5% of the participants referred between 20 and 50% of their diabetes patients to their dental colleagues.

### Knowledge

The knowledge, attitude, and awareness of physicians toward periodontal complications of diabetes patients are summarized in Table 2 . With regard to questions related to the knowledge of periodontal complications in diabetes patients, 289 (84%) of the medical physicians considered “gingival inflammation” and 267 (77.6%) regarded “gingival bleeding” as complications. In addition, 282 (82%) of the physicians considered “periodontal abscess” while 235 (68.3%) considered “alveolar bone resorption” due to periodontal disease as complication in diabetes. Finally, 263 (76.6%) considered “tooth mobility” and 281 (81.7%) considered “tooth loss” as a periodontal complication in diabetes patients ( Table 2 ).

**Table 2:** Items of knowledge, attitude, and awareness toward periodontal complications of diabetic patients

Knowledge		
Periodontal complications	No (%)	Yes (%)
Gingival inflammation	55 (16)	289 (84)
Gingival bleeding	77 (22.4)	267 (77.6)
Tooth mobility	81 (23.5)	263 (76.6)
Periodontal abscess	62 (18)	282 (82)
Alveolar bone resorption	109 (31.7)	235 (68.3)
Tooth loss	63 (18.3)	281 (81.7)
Attitude		
Regular dental checkups are important	39 (11.3)	305 (88.7)
Patients with poorly controlled diabetes should have more frequent dental checkup	41 (11.9)	303 (88.1)
Patients with poorly controlled diabetes should have more frequent scaling	79 (23)	265 (77)
Awareness		
Believe that diabetes affects periodontal health	61 (17.7)	283 (82.3)
Consider that periodontal affects metabolic control	62 (18)	282 (82)
Believe that there is the bidirectional association between diabetes and periodontal health	59 (17.2)	285 (82.8)

### Attitudes

To assess the attitude of medical physicians toward importance of oral health care in diabetes, the survey showed that 305 (88.7%) of the participants believed that “regular dental checkups for DM patients are necessary.” In addition, 303 (88.1%) of the participants thought that patients with poorly controlled diabetes should have more frequent dental checkups, and 265 (77%) of the medical colleagues believed that “patients with poorly controlled diabetes should have more frequent dental scaling” ( Table 2 ).

### Awareness

Regarding the awareness of the participants toward the diabetes-to-periodontitis interrelationship, it was shown that 283 (82.3%) of the participants believed that diabetes affects periodontal health, 282 (82%) of the participants found that periodontal disease affects metabolic control, and 285 (82.8%) of the participants thought that there is bidirectional association between diabetes and periodontal health ( Table 2 ).

The average scores of knowledge for the seven items of “knowledge regarding periodontal complications in diabetic patients” was 4.70 ± 1.38 based on seven questions. With this average, as a cut-off point, imperfect knowledge was reported among 115 (33.4%) and adequate knowledge reported for 229 (65.6%) of the participants. Similarly, the average of knowledge was 4.70 ± 1.38, the average of attitude was 2.54 ± 0.62 based on three questions, and the average of awareness was 2.47 ± 0.74 based on three questions.

### Univariate Analysis for Comparisons of Knowledge, Attitudes, and Awareness by Demographical Data

A cross-tabulation analysis of the available data when comparing knowledge, attitude, and awareness across sectors showed that the proportion of satisfactory knowledge was higher among public sector physicians (69.1%) compared with their private sector colleagues (54.2%) ( *p* = 0.021). No significant difference was found regarding attitude between participants from private and public sectors ( *p* = 0.107). Awareness was higher among participants from public sectors 259 (90.9%) compared with those from private sectors 48 (81.4%; *p* = 0.033). The same analysis, when related to the country of graduation, showed no dependency between the country of graduation and the quality of knowledge, attitude and awareness ( *p* > 0.05; Table 3 ).

**Table 3:** Univariate analysis of association between knowledge, attitude, and awareness by demographical data

Demographics	Knowledge *n* (%)		Attitude *n* (%)		Awareness *n* (%)	
	Poor	Satisfactory	Negative	Positive	Poor	Satisfactory
Practice sector						
Private	27 (45.8)	32 (54.2)	6 (10.2)	53 (89.8)	11 (18.6)	48 (81.4)
Public	88 (30.9)	197 (69.1)	14 (4.9)	271 (95.1)	26 (9.1)	259 (90.9)
* p* -Value	0.021		0.107		0.033	
Gender						
Male	53 (28.6)	132 (71.4)	14 (7.6)	171 (92.4)	17 (9.2)	168 (90.8)
Female	62 (39)	97 (61)	6 (3.8)	153 (96.2)	20 (12.6)	139 (87.4)
* p* -Value	0.028		0.101		0.201	
Country of graduation						
UAE	33 (30.6)	75 (69.4)	4 (3.7)	104 (96.3)	11 (10.2)	97 (89.8)
Others	82 (34.7)	154 (65.3)	16 (6.8)	220 (93.2)	26 (11)	210 (68.6)
* p* -Value	0.262		0.191		0.490	

Abbreviation: UAE, the United Arab Emirates.

When comparing categories of knowledge (poor and satisfactory) with respect to age and years of graduation and experience, there was no significant differences between these parameters while comparing both groups of knowledge ( *p* > 0.05). The same result was obtained for the other items like years since graduation, year of experience, and for the score of awareness. However, the score of attitudes was higher among the group with satisfactory knowledge, 2.59 ± 0.76, compared with a group with poor knowledge, 2.37 ± 0.86, with *p* = 0.029 ( Table 4 ).

**Table 4:** Comparison of categories of knowledge by age, years of graduation, score of attitudes, and score of awareness

Items	Poor knowledge	Satisfactory knowledge	*p* -Value
	Mean (SD)	Mean (SD)	
Age (y)	37.35 (8.39)	38.5 (9.74)	0.287
Years since graduation (y)	12.61 (7.61)	13.90 (9.45)	0.172
Years of experience (y)	11.75 (7.62)	13.32 (9.43)	0.097
Score of attitudes	2.24 (0.71)	2.59 (0.67)	0.029 a
Score of awareness	2.37 (0.86)	2.52 (0.67)	0.085

Abbreviation: SD, standard deviation.

a Statistically significant *p* < 0.05.

### Multivariate Analysis by Using a Logistic Regression Model

A logistic regression model used to explain “knowledge” and possible correlations with various factors (i.e., age, gender, sector, attitude, and awareness of the participants) showed that “gender” is a significant predictor ( *p* = 0.04). The odds ratio (OR) for age (OR = 0.248), however, is less than 1, indicating that as age of the participant increases, he/she is less likely to have adequate knowledge about diabetes-to-periodontitis relationship. With every passing year of increasing age for a physician, the odds of him/her reporting positive “knowledge” decreases by a constant factor (data not presented here), all other factors being equal ( Table 5 ).

**Table 5:** Logistic regression to explain knowledge by possible factors

	*p* -Value	OR (95% CI)
Age	0.248	1.015 (0.99–1.042)
Gender	0.04	0.613 (0.385–0.977)
Sector	0.039	1.882 (1.033–3.429)
Attitude	0.056	2.514 (0.978–6.6.463)
Awareness	0.083	1.884 (0.921–3.856)

Abbreviations: CI, confidence interval; OR, odds ratio.

## Discussion

The survey highlighted the knowledge, attitude, and awareness of medical physicians, practicing within the UAE about the bidirectional relationship of periodontal disease and DM. There is ample evidence that hyperglycemic state in diabetes is associated with adverse periodontal outcomes. 7 10 The consensus report of the European Federation of Periodontology/American Academy of Periodontology (EFP/AAP) workshop on periodontitis and systemic diseases have presented conclusive evidence that patients with severe form of periodontal disease adversely affect glycemic control in diabetes. 10 A dose-dependent cause and effect relationship has been established between the extent of periodontal destruction and complications associated with diabetes. 10 The benefits of managing periodontal disease and its positive impact in controlling diabetes, in terms of reducing HbA1c levels, has been highlighted previously. 19 This strong evidence, derived from various epidemiological studies, primarily suggest that an enhanced communication between the medical physicians and dentists is imperative to achieve adequate resolution of both diseases. 10 This holistic approach toward addressing potential complications of diabetes is also necessary for disease prevention which, in turn, reduces the overall health care burden on the system.

Epidemiological studies that have investigated prevalence of diabetes in this region is sparse. Alawadia et al recently revealed a very high percentage (15.2%) of overall prevalence within the community. 5 In addition, the study also reported percentage of undiagnosed diabetes to be 10% among the UAE nationals and 10.9% in the expatriate population residing in Dubai. This study, not only, concludes an alarmingly high percentage of population suffering from diabetes within the UAE but also highlights the presence of increased number of populations who are unaware of their condition. 5 These numbers are in accordance with the global incidence published in 2019 that one in two (50.1%) of the adults living with diabetes are unaware of their condition. 6 These estimates suggest an urgent global need for improved diabetes screening tools and improved communications between medical physicians and fellow dental colleagues to prevent higher risk of diabetes-related complications. The atlas report published by the International Diabetes Federation in 2019 has also highlighted that the UAE is one of the highest ranked countries in terms of high prevalence of DM. However, only a handful of studies have been conducted to explore primary health care physician's awareness of this interrelationship and clinical relevance.

The American Diabetes Association (ADA) has emphasized the need for comprehensive dental and periodontal examination as a part of the routine initial diabetes care management. 20 Adequate evidence related to this topic has led to the EFP in 2018 to issue consensus report on guidelines for medical physicians and dentists and their patients to encourage early diagnosis, prevention, and comanagement of diabetes and periodontitis. 7 With regard to the knowledge of periodontal complications in diabetic patients, most of the physicians (68–84%) considered gingival inflammation, gingival bleeding, tooth mobility, periodontal abscess, alveolar bone resorption, and tooth loss as possible complications that could be seen in diabetic patients. This is in accordance with other studies that have looked at knowledge and awareness of a different cohort of physicians practicing in Kuwait and Hong Kong. 21 22 A similar percentage of study participants (60–64% for Kuwait and 77.1% of Hong Kong physicians) recognized clinical manifestations of periodontal disease as a complication in diabetic patients. Interestingly, of the 232 physicians practicing in Kuwait who participated in the study, less than 50% of the respondents were aware that tooth loss is a common dental complications among patients with diabetes. This could be construed as an optimistic result in the context that the physicians are aware of some aspects of periodontal diseases that can influence the severity of diabetes, despite minimal understanding of the actual mechanism for such relationship between both diseases.

However, it could also be stated that there is paucity in the physicians' reaction when dealing with diabetic patients. Their awareness about how periodontal complications are commonly seen in diabetic patients, rarely translate to referrals to a dentist or a periodontist for suitable care. Our results highlighted that only 5% of the physicians refer 20 to 50% of their diabetic patients to a dentist which is unfortunate and it clearly reflects a low level of camaraderie between the health care professionals and needs urgent attention. A similar dissociation between awareness and referral practice has been observed among family medicine doctors working in public general outpatient clinic in Kowloon West Cluster of Hong Kong. 22 Although 60% participants demonstrated awareness of the significant relationships between the two diseases, only 12.1% recommended their diabetic patients to see a dentist. 22 Another study conducted in Jordan that looked into physicians' knowledge, perception, and its translation into clinical practice, demonstrated that a relatively optimistic 53% of the doctors referred their diabetic patients regularly to a dentist. 23 However, these results should be interpreted with caution since the study not only included primary health care providers but also endocrinologists practicing in private and community health centers and showed lack of consistency while defining percentage of “regular” referrals. 23 Pertaining to the awareness of physicians toward the diabetes-to-periodontitis interrelationship, this study has shown that most (82–83%) believed that not only does diabetes have a detrimental effect on periodontal health but also periodontal disease affects systemic metabolic control. Thus, the attending physicians depicted superior level of awareness about this bidirectional relationship.

A further in-depth analysis of the available data in this study clearly showed that physicians working in the government-based health care centers had superior knowledge and awareness ( *p* < 0.05 for both these parameters) regarding this correlation than their counterparts in the private sectors. Such results can be attributed to the overall education background of the physicians practicing in different sectors and also an increased exposure to multidisciplinary health care system within the public sector. Analysis regarding knowledge, attitude, and awareness was independent of the physician's country of graduation, highlighting the global nature of diabetes and its complications, in addition to similarities in the level of medical education imparted to the health care professionals. However, a logistic regression model in our study suggests that as age of the participant increases, it was less likely for the professional to have satisfactory knowledge about the diabetes-to-periodontitis relationship. The authors are of the opinion that with increasing age, physicians are less likely to stay abreast with the latest evidence in the field of medicine and dentistry that can influence their knowledge, awareness, and potential attitude toward different subjects in medicine.

## Limitations

The limitations of the present study include complete dependence on data that is self-reported and should be interpreted with caution. The total number of completed surveys may only represent 10% of the total workforce that provides medical treatment in the UAE and hence, the generalizability of the outcomes needs verification. However, it is one of the first survey-based study within the UAE that illustrated the level of knowledge, attitude, and awareness portrayed by medical health care professionals regarding the bidirectional relationship of diabetes and periodontal disease, and how they implement this knowledge in referring diabetic patients for a periodontal examination and suitable dental care to their fellow dental colleagues. A better study design that includes random sampling of all primary health care workers practicing in community-based centers and private sectors alike, may guide in identifying shortcomings in the referral process. Follow-up studies that explore the reasons of nonreferral and possible confounding factors need to be highlighted in future. This can ultimately form the basis for conducting prospective clinical trials that follow diabetic patients over an extended period of time and implications of controlling periodontal parameters for these patients to address this burden on the global health care systems.

## Conclusion

This questionnaire is a preliminary analysis that revealed a huge deficiency among physicians and dentists alike, practicing in the UAE, regarding establishment of a set protocol or guidelines for referring this medically susceptible group of patients for dental care. The survey demonstrated that, although a majority of physicians who treat diabetic patients in their clinical practice present with good knowledge regarding the bidirectional relationship, they rarely refer diabetic patients for dental consultations to receive proper periodontal care. Based on our limited inference, regular and improved communication between medical and oral health care professionals is paramount to ensure comprehensive enhancement of patient's overall health who present with diagnosed or undiagnosed diabetes.
